# Micro-leakage of a Fissure Sealant Cured Using Quartz-tungsten-halogen and Plasma Arc Light Curing Units

**DOI:** 10.5681/joddd.2014.045

**Published:** 2014-12-03

**Authors:** Zahra Bahrololoomi, Ali Asghar Soleimani, Najmeh Jafari, Bentolhoda Varkesh

**Affiliations:** ^1^Associate Professor, Department of Pediatric Dentistry, Faculty of Dentistry, Shahid Sadoughi University of Medical Sciences, Yazd, Iran; ^2^Assistant Professor, Department of Pediatric Dentistry, Faculty of Dentistry, Shahid Sadoughi University of Medical Sciences, Yazd, Iran; ^3^Postgraduate Student, Department of Oral Pathology, Faculty of Dentistry, Shahid Sadoughi University of Medical Sciences, Yazd, Iran; ^4^Postgraduate Student, Department of Pediatric Dentistry, Faculty of Dentistry, Shahid Sadoughi University of Medical Sciences, Yazd, Iran

**Keywords:** Dental leakage, fissure sealants, polymerization

## Abstract

***Background and aims.*** Newer curing units such as plasma arc can polymerize the sealants in much shorter curing times. The aim of this study was to compare the effect of two different curing units on the micro-leakage of a fissure sealant material.

***Materials and methods.*** Sixty two extracted premolars without caries were randomly divided into two groups of 31 samples. Occlusal surfaces of all teeth were cleansed. Then, teeth surfaces were etched by 37% phosphoric acid. After rinsing and drying, occlusal surfaces of teeth were sealed by a fissure sealant. The sealant was then cured using either a halogen light curing unit or a plasma arc curing light. After sealing, the teeth were thermocycled for 500 cycles. The teeth were then sectioned and examined for micro-leakage. Statistical analyses were performed with Mann-Whitney test.

***Results.*** There was no significant difference between two groups regarding micro-leakage (P = 0.42).

***Conclusion.*** Results showed that there was no significant difference between two different curing units. Therefore, plasma arc unit might be a useful alternative for sealant polymerization.

## Introduction

In spite of numerous prevention methods, dental caries is still a highly prevalent disease in the world.1 Placement of sealant is considered a highly effective method for prevention of carious lesions in occlusal pits and fissures.2 The preventive benefits of this treatment method is related to the ability of the resin sealant to fill pits and fissures and remain intact and bonded to enamel for lifelong.^1^

Quartz-tungsten halogen curing units (QTH) are commonly used for sealant polymerization, because they are inexpensive and well established.^3,4^ However, the high intensity of Plasma arc curing (PAC) light source allows the composite materials to be cured much faster than conventional lights;^5^ although shorter curing times may lead to inadequate polymerization and increased micro-leakage along the dentin margins and early failure in direct resin composite restorations.^6-7^

The effect of shorter curing time with high power sources remain controversial.^8^ Shah et al^4^ found no difference regarding micro-leakage between resin-based sealants polymerized with QTH or PAC; but in the study conducted by Jacinta et al,^7^ the use of PAC light curing in continuous or step-cycle modes resulted in increased micro-leakage.The purpose of this study was to evaluate the effect of plasma arc curing unit in comparison with QTH source on the micro-leakage of sealants.

## Materials and Methods

Sixty two intact premolars extracted for orthodontic reasons were included in this *in vitro* study. The teeth were free of cracks, caries and restorations. Periodontal curettes were used for removal of remnants of soft tissue. Prior to the study, the occlusal surfaces of the teeth were cleansed with water/pumice slurry using brushes at low speed. The specimens were randomly divided into two groups (n = 31).

Initially, the specimens were gently air-dried. The enamel was acid-etched using 37% phosphoric acid (3M-ESPE, St. Paul, USA) for 15 seconds. Air and water sprays were used for 10 seconds to completely rinse the acid and dry the teeth. Then the fissures of teeth were sealed with Clinpro sealant (3M-ESPE, St. Paul, USA). In group I, the sealant was cured using a halogen light cure (Arialux, Apadana Tak, Iran) with 500 mW/cm^2^ intensity for 20 s. In group II, curing was done with plasma arc curing light (LiTey 685, Dent, America) with 1200 mW/cm^2^ intensity for five seconds.

After curing, the teeth were thermocycled 500 times at 5 ± 2°C to 55±2°C. The surfaces of the specimens were coated by two layers of nail polish except for one millimeter around the sealant. The specimens were stored in 2% methylene blue for 48 h, then were rinsed and sectioned bucco-lingually in mesial and distal surfaces of each tooth (3 sections) to assess dye penetration under ×15 magnification of a stereomicroscope (sten SV 11, Zeiss, Germany).

Three observers assessed the micro-leakage, and the results after their agreement were recorded. A ranked scale was used to score dye penetration (micro-leakage) as follows:

0: no dye penetration

1: dye penetration limited to the outer half of the sealant

2: leakage up to the inner half of the sealant

3: dye penetration extending to the underlying fissure

Data were analyzed by SPSS (ver. 18) using Mann-Whitney test.

## Results

Table 1 shows the comparison of micro-leakage (dye penetration) score between two different methods in tooth sections. Statistical analysis with Mann-Whitney test showed no significant difference between two groups (P = 0.22).

**Table 1:** Comparison of micro-leakage between two different methods in tooth sections evaluated (n = 124)

Curing light unit	Mean ±SD	Median	Dye penetration score							
			0		1		2		3	
			n	%	n	%	n	%	n	%
Halogen	0.88±1.2	0	76	61.26	8	6.45	19	15.32	21	16.93
Plasma arc	1.04±1.32	0	65	52.41	12	9.61	23	18.54	24	19.35
Micro-leakage score: 0: no dye penetration; 1: dye penetration limited to the outer half of the sealant; 2: leakage up to the inner half of the sealant; 3: dye penetration extending to the underlying fissure										

Dye penetration was seen in 61.27% (n = 19) and 67.73% (n = 21) of specimens in the halogen and plasma groups, respectively (Table 2). Comparison of teeth with the same test showed that there was no statistically significant difference between the two groups (P = 0.42).

**Table 2:** Comparison of micro-leakage between two different methods in the specimens evaluated (n = 31)

Curing light unit	Mean ±SD	Median	Dye penetration score							
			0		1		2		3	
			n	%	n	%	n	%	n	%
Halogen	1.32±1.24	1	12	38.7	5	16.12	6	19.35	8	25.8
Plasma arc	1.58±1.28	2	10	32.25	4	12.9	6	19.35	11	35.48
Dye penetration score: 0: no dye penetration; 1: dye penetration limited to the outer half of the sealant; 2: leakage up to the inner half of the sealant; 3: dye penetration extending to the underlying fissure.										

## Discussion

Fissure sealant was introduced to prevent occlusal caries more than 30 years ago.^1^ Since then, fissure sealant application gained increasing utility, and its efficacy was proved in many studies.^9,10^

The caries reduction ability and effectiveness of a fissure sealant are closely related to the integrity of enamel-sealant interface and subsequent sealant retention.^1^ Various types of curing lights have been introduced for photo-polymerization of resin composite, naming some of them: conventional QTH, light-emitting diode (LED), PAC and laser curing lights.^6^

One of the methods introduced to increase the power density is application of high intensity PAC light unit. The advantages of plasma arc curing lights with their shorter curing times, in patient management, have made them a beneficial tool as a treatment modality in children. In addition to its beneficial characteristic in the management of anxious and uncooperative patients, reduction in operating time is beneficial to both patient and dentist.^4^

Polymerization shrinkage is an important issue as it affects the marginal seal and subsequent integrity of the restoration. There are several techniques for assessment of micro-leakage around dental restorations. Dye penetration is a common method in orthodontic and restorative dentistry, which includes exposing the samples to a dye solution and then, viewing cross-sections under a light microscope.^5,11,12^ In the current study, we used this technique because of its simplicity and low price. Also in this study three sections (four surfaces) from each tooth were evaluated, which improves its accuracy.

The findings of this *in vitro* study indicated that dye penetration was seen in both groups. Statistical analysis showed no significant difference between two groups regarding micro- leakage. Most studies have assessed the effect of plasma arc on composite resins, compomers or resin-modified glass-ionomers.^2,13^ There are few studies conducted on the effect of this method on sealants.^4^

Shah et al^4^ demonstrated no significant difference in the degree of micro-leakage of sealants polymerized by a conventional QTH curing light compared to a PAC light.

While polymerization of composite resin with a plasma arc unit resulted in an increase in dye penetration along the resin-tooth interface compared with QTH curing light, none of the light sources seemed to have a significant effect on the sealant micro-leakage.^4^ Composite resins have a higher filler load compared to unfilled sealants, which may justify a higher stress build-up at the tooth-restoration interface,^4^ and explain why the sealants may have less micro-leakage than composite resins.

Uysal et al^12^ have demonstrated that bands cemented with PAC had significantly higher amount of micro-leakage in comparison to LED and QTH at the cement-enamel interface.^12^ Davari et al^14^ in a study for assessment of the microleakage under ceramic and metal brackets bonded with LED and plasma arc curing unit found that LED units lead to more microleakage comparing plasma arc units.^14^

Plasma arc curing affects the micro-leakage of class V resin-based composite restorations. Some studies on polymerization of composite resin by these units have identified other concerns, such as temperature generation and incomplete polymerization.^3^ Oztuk et al^15^ postulated that plasma arc units are not to be used in deep cavities. A high intensity output plasma arc curing light probably leads to reduction of the curing time. Insufficient irradiation time may cause incomplete monomer conversion and composite polymerization.^13^

Composite resins cured with plasma arc unit may show greater water solubility in contrast to materials cured with conventional light units. This is probably not important in polymerizing sealants with the PAC light source, since the thickness of the sealant is reduced in comparison with composite resin restorations.

The results of this study are promising for the use of PAC light unit in sealant curing. This will save time and would be useful to busy clinicians.

## Conclusion

Within the limitations of this laboratory study, it is concluded that plasma arc curing light may be a useful alternative in sealant polymerization.

## Acknowledgement

The authors are grateful to the staff at the faculty of dentisty, who assisted the authors in this project.
